# Curiosities of Medical Literature

**Published:** 1848-11

**Authors:** 


*Curiosities of Medical Literature.*—Medical literature, bibliographical and periodical, occasionally offers some curious specimens. The idea occurred to us, some time ago, of collecting some choice *morceaux* under the title which we have prefixed to this article; but, through inadvertancy it has been suffered to lie dormant. The thought has been revived by perusing a communication contained in our respected contemporary, the Boston Medical and Surgical Journal, for Oct. 18th, 1848. The burthen of the communication is an account of a case of supposed vesical calculus, in which the operation of lithotomy demonstrated an error of diagnosis, a mistake which, we believe, some excellent surgeons have been known to fall into, without any cause for imputation of unskilfulness, and, certainly, without furnishing an occasion for an insinuation against surgery in general, which appears to be the object of the author of the communication referred to. But the *curiosities* in the communication are contained in the following paragraph:—"If I may be permitted to digress, this, and several other cases, have somewhat shaken my credence in the powers of auscultation by practitioners *generally*. A dearly esteemed friend of mine recently died. His disease was a softening of the substance of the brain *extending* to suppuration, which his stethoscopic examiners (eminent men) attributed to, and treated, for seven years, as a *diseased liver!* A *post mortem* examination demonstrated that this organ, with the abdominal and thoracic viscera, were in a perfect state. * * * Two years' zealous application to the study of the use of the stethoscope and auscultation, with a good "ear" for music, too, enabled me to distinguish for a "burring" sound in the left lung (the side upon which I had my ear) the rolling of a fire engine down the street."

The communication from which the above remarkable paragraph is quoted, has the signature of A. C. Castle, M. D., Surgeon Dentist, New York. We give the name, so that the important information enunciated in the quotation may have the full benefit of it. Dr. Castle has not much confidence in the "powers of auscultation by practitioners *generally*," because an esteemed friend died of softening of brain (*extending* to suppuration!) which had been treated as a diseased liver!! It is to be regretted that the learned doctor did not amplify upon the subject, and inform us what particular relations exist between the "power of auscultation," and softening of the brain, or diseased liver. It strikes our poor apprehension that it would have been somewhat more apposite if the Doctor had quoted a case in which an error of diagnosis relating to pulmonary or cardiac disease had been committed. We are also (in our stupidity) at a loss to perceive the necessity of a digression concerning the "power of auscultation" in a case of supposed calculus of the bladder. But the confession contained in the last sentence is the most curiously interesting. The Doctor, it seems, applied himself zealously for two years to the study of the stethoscope and auscultation, with a good ear to boot, and was only able to distinguish the sound of a fire engine rolling down the street as a "burring" which, he had at first referred to the chest! Where in the whole range of philology did the term "burring" come from, as applied to an auscultatory sign! The reader will, assuredly, not wonder that the Doctor has but little confidence in the "powers of auscultation!" The confession shows that a "good ear for music" does not alone suffice for a good auscultator, but that some brains in addition are required. It must be admitted that the pen is a better instrument for revealing cerebral softening than the stethoscope.

